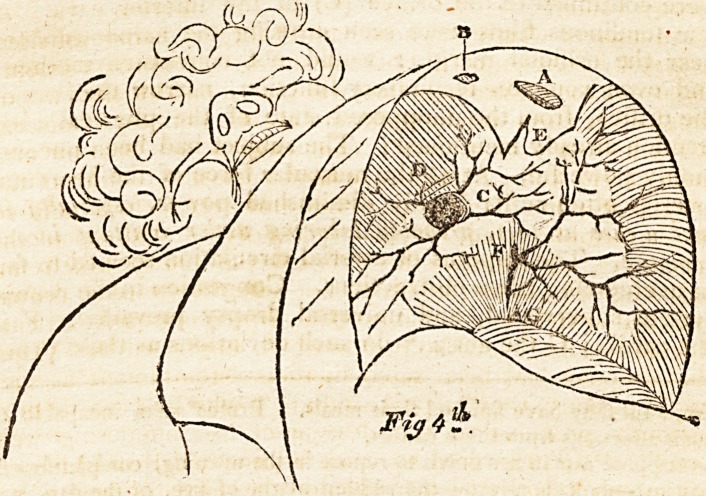# Observations on the Vis Insita Musculorum, the Laws of Which Seem to Have Been Overlooked, in Some Measure, by Natural Philosophers, in Explaining Muscular Attachments and Their Phenomena

**Published:** 1814-04

**Authors:** Alex. Ramsay

**Affiliations:** 3, Villa Place, Walworth Common, near London


*For the Medical and Physical Journal.*

### *Observations on the Vis Insita Musculorum, the Laws of which seem to have been overlooked, in some measure, by Natural Philosophers, in explaining Muscular Attachments and their Phenomena; by* Dr. ALEX. RAMSAY.

ACCORDING to the rules of applying moving powers to levers where dead organs are the forces employed, natural philosophers argue correctly in asserting, *that the human muscles, by subtending small angles at the hinge or moving extremity of joints, are disadvantageously situated.*

If I am correct, however, in my opinions respecting the vis insita musculorum, the extension of the angle, *or in other words extending the moving power toward the remote extremity of the lever*, would promote a more objectionable inconvenience.

The muscles of the vital organs seem to possess one power, and only display one physical phenomenon, i. e. the vis insita musculorum. All these organs may, *by way of distinction*, be named hollow muscular agents; they are peculiar recipients; their contents at the same time distend, and excite their muscles to contraction. These muscles are for the most part annular or spiral; they are not opposed by other muscles; only a few individuals of men are on record who have possessed either power over, or cognizance of, these organs.

The muscles of locomotion present to our consideration very different phenomena. They likewise possess the vis insita musculorum, but we distinguish opponent muscles annexed to every member. Over all these the soul has power; and of all these, this immortal being has cognizance: hence, in the muscles of locomotion, we contemplate the wonderful superinducement of mental influence. By a few observations *on the influence of the vis insita, its connection in man with a large brain, and the miraculous force which mental influence has in promoting power*, perhaps we may in some measure account for the mode of muscular insertions, as a strong demonstration of creative wisdom.

*The Vis insita musculorum, &c. considered.*—Contraction *to a certain extent* seems the physical action of muscles, perfectly independent of mental influence, which indeed does not enter in any degree into the cause of pure physical phenomena; this is illustrated in the attitudes physically assumed by some organs. If the anatomist attends to a person standing before him, he will discover the hand, when carelessly lessly hanging by the side, assumes the attitude Fig. 1st, i. e. the hand, is in the position, between complete pronation and supination, the thumb nearly touching the thigh.

*Fig.* 1*st.* *Fig.* 2*d.* *Fig.* 3*d.*

When the soul is directed to the supinators, the superinduced mental influence of the supinators overcomes the vis insita of the pronators, and the hand performs half a rotation, as in fig. 2d, presenting a full supine view of the palm. When we attend to the phenomena exhibited in active motions, we seem entitled to suppose, that in consequence of every active exertion of one set of muscles, superinduced by mental influence, not only is the organ exhausted which is immediately influenced by the soul, but the opponent is exhausted likewise by operating against the active organ, by its vis insita or physical contractile power, ever present, ever acting. We cannot seemingly account for the stability of animal motions, but upon this principle. Were only the muscles which occasion flexion to exert, the limb must act by a jerk, unsteady and irregular, as in a dead body; or such as is exhibited in a paralysed member, which instantly starts by the influence of soul on the more living muscles, which not being balanced by opponent vis insita, we discover the half willing, half unconscious, operations of this disease. If I am correct in these statements, we account for the fatigue of even a stationary erect posture, as the opponent muscles of the trunk and inferior extremities are called into incessant exertion of their vis insita, in balancing the perpendicular figure of man. Brutes suffer comparatively little fatigue in standing, their base of support is so extended. If the reader attends to a person in an erect stationary attitude for any time, by directing the eye on his figure, *especially the head,* and comparing this head with a fixed distant object, he will be convinced that the person who stands,

 though though seeming to stand firm, is really continually in a rolling motion: one moment the flexors of the toes prevent him from falling off the centre, and another moment the posterior muscles correct physical danger, and toss him upon his centre. All this goes on without his consciousness: they are physical operations, excited and corrected by physical innate laws. Our system seems so wisely formed, as to carry on every function that is possibly necessary by laws planted in the system of our organs, by the Creator, perfectly independent of our exertions, leaving nothing for us to do but to use a perfect and unincumbered series of machinery. The reader attending to fig. 3d, perceives the hand in a complete state of pronation, which, compared with fig. 2d, conveys a notion of the whole range of palmar rotation, fig. 2d and 3d being the consequence of the utmost mental influence upon the rotation of the radius by its muscles. Fig. 1st represents the physical equilibrium of the vis insita of the supinators and pronators, which, left to themselves, by pulling equally, the hand falls into the semiprone state.

May I presume, in supposing, that our attention to the hand, in its physical attitude in another sense, seems to favor the notion of the vis insita in its influence? by adverting to the three figures of the hand, *which are taken from a strong man*—the fingers assume a bent position. This, though the hand of a gentleman, whose muscles are not so contractile as those of a laborer, never naturally appears more straight, unless the mind is directed to the extensors; and very powerful laboring people exhibit their hand nearly half closed, when it assumes its physical attitude. The flexors are very powerful; hence their proportionate vis insita occasions this attitude. A laboring man cannot bring his hand to a plain surface. That the extensors are merely sufficiently powerful to open the hand, and thus the vis insita of the flexors is not needlessly exhausted in closing the fingers, surely claims from us, *the rational offspring of a God*, the tribute of adoration and gratitude; and the reader would have reason to blame an author, did he not lead others as well as himself to the improvement of our knowledge of the ways of Providence in the last and most perfect work of creative munificence—Man!

As this is an age full of argument, *but chiefly only on one side, argument against sober opinions; to philosophise truly, we should have no side, no party; but bring forward data, and reason à posteriori: there is no end or benefit in theorizing à priori.* For the sake of argument, let us suppose that one of our philosophers in Paris, should make his perfect man. Let his biceps brachii reach to the carpal extremity of the the radius, and the brachialis internus be inserted into the carpal end of the ulna. This lever has great power; but since its physical contraction is operating, *that the fore-arm may proceed in the contrary direction*, what is gained, since the extensor must correspond? Here is an immense deformed Parisian arm: the quantity of living vessels, muscles, and nerves, thus impressed upon a common brain, must surely unhinge the sensorium commune. But we must, perhaps, have likewise a Parisian new-framed sensorium, &c. and French chemists have made more bold attempts than this. How much more wisely has the God of Nature done! who, knowing the power of soul in kindling up instant and miraculously-powerful contraction, has used as little active matter as possible. We may easily convince ourselves that the exertion of soul alone can command this power, as powerful feats are performed either by sudden jerks, or steady and continued direction of the soul to the muscles exerting. We cannot calmly produce powerful contraction. Energy, anger, eagerness, desire, &c. must direct velocity or power. May I presume, that while we seem convinced of the existence of physical contraction, and contraction by mental influence, neither physical or mental causes can produce relaxation? When the tendo achillis is snapped, or the patella fractured, the muscles instantly assume their complete physical contractility; the same takes place in paralysis. The vis insita does not relax them. The mind has no influence on these muscles, when it ceases to have a power of restoring them by their appropriate opponents; relaxation is always a forced state by the medium of a foreign agent or opponent. So in the vital organs, their muscles are forced to their relaxation by their contents, as well as induced to contraction by the stimulus of their distending contents. This process, *if I am in an error, your learned correspondents will put me right*, seems a divine simplicity of one cause, producing two opposite consequences. The muscles yield, till the stimulus of distention force them to the convulsion of contraction.*

It

* Am I correct in supposing that the humerus must be luxated continually, did not the stimulus of the muscles, which contract by mental influence, excite a corresponding ratio of physical contractility in the opponent muscles? If the person who reads this try the experiment, he will be convinced, that while he clinches his fist, the extensors are excited in regulating the motion at the same moment. Hence I always recommend reducing luxation by previous syncope, induced by warm bath, or means of tobacco, &c. as nature revolts at

It may not, I trust, be thought an irrelevant observation, that the vital organs are rarely paralysed, while those actuated by soul are very obnoxious to this malady. Brutes are by no means so liable to palsy as man; does this depend on their being more merely physical machines? Man seems to possess, *in proportion to his muscles, in consequence, perhaps, of his power of mind,* more physical power than any other animal. Your readers may probably recollect, not only the following, but more remarkable instances of human power, as well as ancient brutality, which sacrificed every thing to its own vanity.

An ancient warrior (whose name I forget) being solicited to give a specimen of his herculean force, before an assemblage of heroes, a strong helmet was placed on a block of wood, which was to be cloven by the sword of this exhibitor. Looking at the helmet with a steady countenance, his brawny arm hung carelessly by his side, his hand enclosing the hilt of the mortal weapon. He was observed, however, before he lifted his irresistible hand, to look furiously around upon the spectators, after which he inflicted the blow, which not only cleft the helmet in twain, but left the sword so deeply enclosed in the block, that no person present could extricate it from the wood but the power which engrafted it. When the company had been sated in their astonishment at such might of body, they began to inquire "why he eyed them so furiously previous to the descent of his thundering weapon?" "I," answered he, "determined, that if I failed in my attempt, no living man should relate my misfortune."*

Having

---

at our levers and torture, in pulling a poor wretch to pieces. The celebrated Dr. Cullen used to give us some wholesome lessons respecting nurses and nature. "Never," said he, "speak of the power of your medicines before the nurse: perhaps she knows she never administered them." And Nature smiles at us behind the curtain occasionally, as in the following instance. A poor man in the Edinburgh Hospital had been put on the rack for a luxation of the shoulder joint: the surgeons were foiled—the man was put half dead to bed—the system was completely relaxed—he fell asleep—kind nature left to herself, the opponent muscles merely regulated gentle motion; they were no longer excited to rebellion by application of surgical skill. The man was found by the attendants fast asleep, his arm thrown over his head, and the os humeri in its place.

* I shall venture another consideration, which may, by the judicious practitioner, be often rendered useful in practice. I often employ the mind of patients in treating their maladies; and through this solitary medium have kept people alive for days in comfort—where soul is concerned in the promotion of disease or health, and where we

Having already extended this paper to a great length, I shall at present make but one further observation.

I annex a diaphragmatic diagram (fig. 4th) as one of the innumerable specimens of structure of animal organs, where the Divine Being teaches us two grand lessons—1st, That the harmonious and perfect manner usually directed by his *sacred finger is direction,* and not a necessary and undeviating law. 2dly, These deviations demonstrate to us, by absolute contrast, the infinite wisdom and goodness of those modifications usually observed by our Maker.

*Fig 4th.*

The subject from which this diagram was taken, came before me in the years 1792–93, when I visited Dublin as a medical student.

---

we can in some degree distinguish the intervening link between human and brutal intellect.

There seems some inherent power in the muscular system, by which means physical exhaustion is instantly regenerated or evolved on some occasions; and on other occasions, physical power is instantly extinguished for ever. *The modus operandi of any one phenomenon shall probably ever elude human research, at least until philosophy is in better hands than those who have put true philosophising completely to the blush, and at a stand, by setting Bacon and Newton, as well as their God, out of sight.*

When we sit down to dinner with a hearty appetite, sudden good or bad news shall instantly banish hunger as effectually as repletion. We often account for this from a slight nausea and anorexia, flatus, and flutter of the pulse: it seems a feverish rouzing of the heart and the gastric system. I find that lively children are equally susceptible of this false gastric loss of craving: I therefore avoid rousing their fancy student. His age seemed 45. Every department of the system exhibited complete dropsy. Upon opening the abdomen, the following circumstances were observable. At A, the diaphragm was not connected with the ribs; the peritonæum run upward into the thoracic cavity $2\frac{3}{4}$ of an inch, the longest diameter was $1\frac{3}{4}$ of an inch. A similar one on the right side of the ensiform cartilage is represented in perspective at B. I have here left part of the peritoneal expansion (E), the better to illustrate the fact. The cordiform tendon of the diaphragm was interrupted at C. The muscular fibres (D) were continued to the orifice (C) of the inferior cava. A few tendinous films were seen crossing the carneous fibres near the orificial margin: would not convulsive motions, and even common respiratory function, narrow the area of the orifice, from the unopposed state of the physical contraction already mentioned? The subject had been uncommonly powerful. At 45 the muscular force of the heart and arteries often suffer highly diminished power, *especially in such a case as this, probably laboring under constant inconvenience.* The impetus of arterial circulation seemed to fail in overcoming these obstructions. Congestion in the venous system supervened, and universal dropsy prevailed. F is the cardia; G the aorta:* do such deviations as these prove

---

fancy till they have finished their meals. Brutes seem incapable of such affections from these causes.

Suppose a man lies down to repose in the evening, completely exhausted—he falls asleep—the sudden alarm of fire, or the dark lanthorn of the assassin, shall, in the twinkling of an eye, conjure up infuriate energy—every muscle is strung; nature no longer conveys cognizance of weariness to her immortal inhabitant; he rises the same as if regenerated by balmy sleep, consummated by all the auxiliaries of nocturnal digestion and rest.

With difficulty the affrighted warrior, in the midnight onset, rouses his faithful horse: on these occasions, his brutal intellect receives no informations, his animal economy no renovation. The child is often deranged by nocturnal frights, from absolute apprehension, without absolute experience of events.

* As several of my philosophical friends, *not of the profession,* are subscribers to your valuable Journal, I may mention, for their information, the philosophy of the diaphragmatic structure, as it usually appears. The inverted cardiform portion, *of which C forms the centre,* in the diaphragm, in usual cases, is tendinous, the tendon reaching upward to D. By this structure, *as tendon is a passive substance, at least does not contract, though it may admit a little dilatation,* the muscles which surround the tendon, *in the act of contracting,* equally dilate a little the venal orifice (C); but in the case before us, the muscles (D) being continued to the orifice, their contraction will narrow, and in a measure obliterate the orifice.

to to us the wisdom of construction? Do the usual hidden occurrences, developed by the anatomist, illustrate the slender, but sure and gradual premature mortality of human nature?

ALEX. RAMSAY, M.D.

*3, Villa Place, Walworth Common, near London, March 2,* 1814.

## Figures and Tables

**Fig. 1st. f1:**
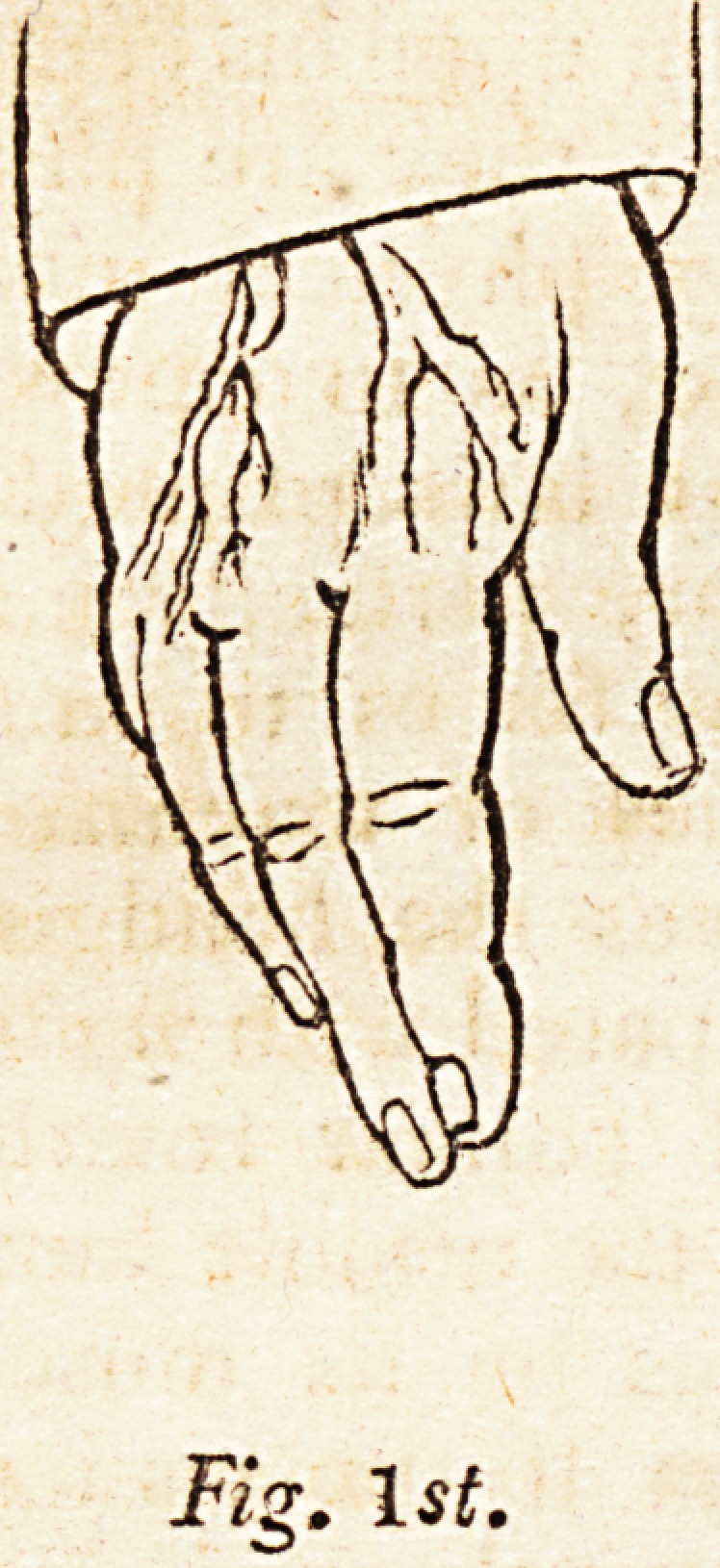


**Fig. 2d. f2:**
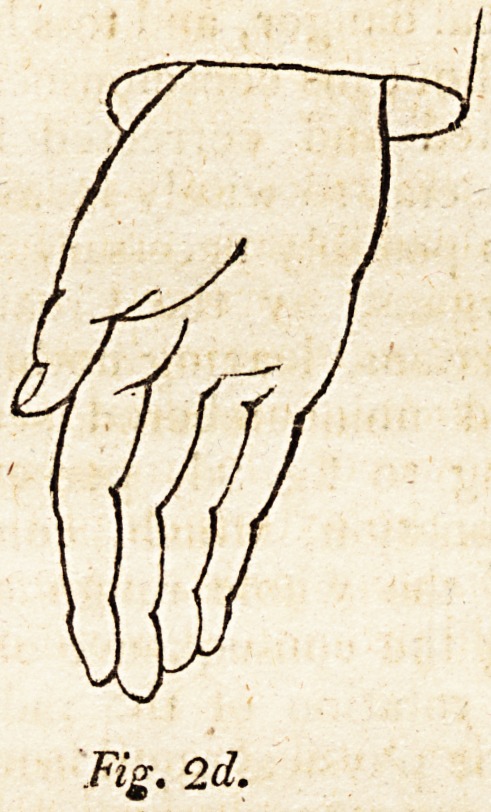


**Fig. 3d. f3:**
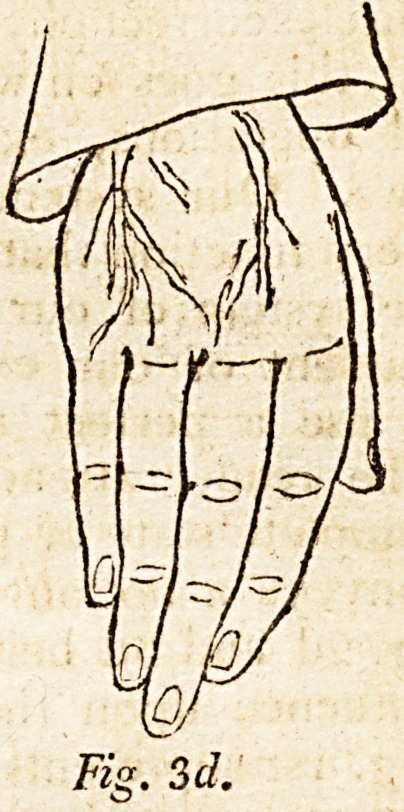


**Fig 4th. f4:**